# PTV margin for dose‐escalated radiation therapy of prostate cancer with daily online realignment using internal fiducial markers: Monte Carlo approach and dose population histogram (DPH) analysis

**DOI:** 10.1120/jacmp.v7i2.2210

**Published:** 2006-05-25

**Authors:** Miao Zhang, Vitali Moiseenko, Mitchell Liu

**Affiliations:** ^1^ Department of Medical Physics British Columbia Cancer Agency‐Fraser Valley Centre 13750 96th Avenue, Surrey British Columbia V3V 1Z2 Canada

**Keywords:** Patient realignment, organ motion simulation, prostate cancer dose escalation, dose‐population histogram, biological factors

## Abstract

Using internal fiducial markers and electronic portal imaging (EPI) to realign patients has been shown to significantly reduce positioning uncertainties in prostate radiation treatment. This creates the possibility of decreasing the planning target volume (PTV) margin added on the clinical target volume (CTV), which in turn may allow for dose escalation. We compared the outcome of two plans: 70 Gy/35 fx, 10‐mm PTV margin without patient realignment (Reference Plan) and 78 Gy/39 fx, 5‐mm PTV margin with patient realignment (Escalated Plan). Four‐field‐oblique (gantry angles 35°, 90°, 270°, 325°) beam arrangement was used. Monte Carlo code was used to simulate the daily organ motion. Dose to each organ was calculated. Tumor control probability (TCP) and the effective dose to critical organs $\left(D_{\text{eff}}\right)$ were calculated using the biologically normalized dose‐volume histograms. By comparing the biological factors, we found that the prescription dose can be escalated to 78 Gy/39 fx with a 5‐mm PTV margin when using internal fiducial markers and EPI. Based on the available dose‐response data for intermediate risk prostate patients, this will result in a 20% increase of local control and significantly reduced rectal complications provided that less serial dose‐volume behavior of rectum is proven.

PACS number: 87.50.‐a

## I. INTRODUCTION

Planning target volume (PTV) margins are conventionally added to the clinical target volume (CTV) to account for uncertainties associated with organ motion and day‐to‐day setup variation. These margins can be uniform or asymmetric, depending on organ motion and risk of toxicity to surrounding organs. There appears to be a certain degree of variation among cancer centers regarding the prostate PTV definition.^(^ ^1^ ^–^ ^6^ ^)^ Commonly, the margin is approximately 1 cm with a smaller posterior margin to achieve better rectal sparing. A more generic approach has been suggested by van Herk,^(7)^ who proposed linking the PTV margin with systematic and random errors.

PTV margins can be reduced if better tumor targeting is achieved, for which various methods have been suggested. Patient realignment alone is adequate if interfraction motion is considerable while intrafraction motion is not as significant, for example, the prostate. This is in contrast to gating techniques, when intrafraction motion is significant, for example, the lung. Realigning the patient^(8)^ allows us to reduce the PTV margin while keeping the CTV adequately covered, sparing normal tissues and potentially escalating the dose.

In our previous work,^(9)^ we tested PTV margins from 2 mm to 12 mm using three real patient anatomies with full (no realignment) or reduced (daily online realignment) uncertainties. An online realignment^(8)^ using fiducial markers implanted in the prostate, in combination with daily electronic portal image (EPI) acquisition prior to each fraction, was simulated. We assumed that only lateral images were taken to localize the prostate, following the Princess Margaret Hospital protocol.^(8)^ Of all the considered margins, 4‐mm and 6‐mm margins were deemed acceptable for dose escalation up to 74 Gy or 78 Gy while keeping rectal toxicity at the level associated with the larger PTV and without realignment (full uncertainties).

In this paper, we extend this study to ensure that these smaller margins will be applicable to a larger group of patients. We also specifically address the importance of dose‐volume effects in the rectum for dose escalation with patient realignment. This topic has become debatable recently because evidence pointing toward a more parallel behavior^(^ ^9^ ^,^ ^10^ ^)^ for rectal toxicity has been presented. This is in contrast to previously assumed serial behavior.^(^ ^11^ ^,^ ^12^ ^)^ There may also be a difference in the volume dependence, depending on the severity of the rectal toxicity.

## II. METHODS

### A. Treatment planning

The purpose of this study was to investigate whether PTV margins can be safely decreased, in order to perform dose escalation. The CTV was defined as the prostate; seminal vesicles and pelvic lymph nodes were not included. The rectum and bladder were considered organs at risk (OARs). CT scans of 20 prostate cancer patients were included in the study. The prostate was contoured by the treating radiation oncologist. Both the bladder and rectum were contoured as solid organs. The rectum was contoured from the anal verge to the level where it becomes the sigmoid colon. Treatment planning was performed on the Eclipse treatment‐planning system (Varian, Palo Alto, CA) for a Varian EX 120 multileaf collimator (MLC) linear accelerator. Three‐dimensional uniform margins of 10 mm and 5 mm were automatically added to the prostate (CTV) to obtain the PTVs. In this paper, a 10‐mm PTV margin with a prescription dose of 70 Gy/35 fx and no patient realignment is referred to as a Reference Plan. A treatment plan with a 5‐mm margin with a prescribed dose of 78 Gy/39 fx with patient realignment is called an Escalated Plan.

The beam arrangement was a four‐field setup, using two lateral and two anterior‐oblique fields with gantry angles of 35°, 90°, 270°, and 325°.^(1)^ The MLC leaf positions were manually adjusted to cover the entire PTV with the 95% isodose surface.

### B. Organ motion simulation and dose calculation

A Monte Carlo code running in the MATLAB (MathWorks, Natick, MA) environment^(13)^ was developed to simulate geometric uncertainties during the treatment on a fraction‐by‐fraction basis. The code simulates organ motion with the following assumptions: (1) organ deformation and rotation are not considered, and (2) relative positions between the prostate, rectum, and bladder do not change. Geometric uncertainty was separated into systematic and random elements, which were assumed to obey a Gaussian distribution.^(7)^The overall displacement of a single fraction was modeled as a sum of systematic and random components. For a specific virtual patient, the systematic uncertainty was sampled once and was kept constant for each fraction through the course of treatment. Random uncertainty was sampled for each fraction.

The standard deviations associated with both systematic and random uncertainties used in this simulation were taken from the published literature (Table 1. For the treatment without patient realignment, that is, the full uncertainty situation, the standard deviations were taken from Table 1 of the paper by van Herk et al.^(7)^ The reduced uncertainty data (i.e., after online correction with a 3‐mm action level) were taken from Table 3 of the paper by Chung et al.,^(8)^ the “center of mass” data. Two hundred histories were calculated for each treatment plan with a calculation time of 6 h to 7 h on a Pentium IV 2.66 GHz PC. This number of histories was demonstrated to be sufficient to establish confidence limits within a reasonable duration of calculation.

**Table 1:** The standard deviations of geometric uncertainties used in the simulations

	Systematic (mm)			Random (mm)		
	SI	AP	Lat	SI	AP	Lat
full uncertainly^(7)^	3.6	4.1	3.2	2.5	3.2	2.2
reduced uncertainty^(8)^	0.6	0.5	3.2	1.1	1.2	2.2

The dose distribution from the treatment‐planning system was used to calculate the dose delivered every fraction. In the following sections, any results referred to as “static” were calculated without organ motion simulations; for example, the dose to each organ is the same as the planning dose for each fraction. If the dose distribution or a specific parameter is referred to as “blurred,” this means that the results were obtained with organ motion simulation.

Dose from each fraction was accumulated on a voxel‐by‐voxel basis for each organ.^(^ ^14^ ^,^ ^15^ ^)^ For each voxel, dose was converted to the normalized total dose (NTD),^(16)^ which is the biologically equivalent dose in 2 Gy/fraction. The formula used to convert dose to one voxel for the *i*th fraction to NTD is

(1) $${\text{NTD}}_{i}\frac{d_{i}[\alpha /\beta +d_{i}]}{\alpha /\beta +2\text{Gy}},$$

where α and β are the parameters in the linear‐quadratic model. The values are listed in Table 2.

**Table 2:** Parameters used in biological factor calculation^(^ ^11^ ^,^ ^12^ ^,^ ^17^ ^–^ ^19^ ^)^

	$D_{50}$	α/β	$\gamma_{50}$	*n*
TCP	66.8 Gy	1.5 Gy	2.3	
bladder $D_{\text{eff}}$	80 Gy	3 Gy		0.5
rectum $D_{\text{eff}}$ (serial)	80 Gy	3 Gy		0.12
rectum $D_{\text{eff}}$ (parallel)	56.7 Gy	3 Gy		0.746

### C. Biological factor calculation

Each plan was evaluated for both local control and toxicity. This was done by calculating biological indices: TCP^(^ ^17^ ^,^ ^18^ ^)^ and $D_{\text{eff}}$ (isoeffective dose to whole volume).^(^ ^11^ ^,^ ^12^ ^,^ ^19^ ^,^ ^20^ ^)^ Note that the TCP has to be defined for the gross tumor volume (GTV); however, the GTV is equivalent to the CTV in the case of prostate cancer. If the whole CTV receives a uniform dose $D_{i}$, then the TCP can be calculated as

(2) $$\text{TCP}(D_{i})\frac{1}{1+\exp[-4\gamma_{50}(\frac{D_{i}}{D_{50}}-1)]},$$

where $D_{50}$ is the dose leading to a 50% probability of local control, and $\gamma_{50}$ is the normalized slope of the TCP versus dose curve at 50% TCP.^(^ ^17^ ^,^ ^18^ ^)^ The probability to control a voxel with a volume $v_{i}$ receiving dose $D_{i}$ is

(3) $$\text{TCP}(v_{i},D_{i})=\text{TCP}{\left(D_{i}\right)}^{v_{i}}$$

To control the whole CTV, all voxels have to be controlled; therefore, the overall TCP for the CTV is

(4) $$\text{TCP}=\Pi \;\text{TCP}(v_{i},D_{i})$$

For the critical organs, the $D_{\text{eff}}$ was calculated using a power‐law‐based algorithm^(^ ^19^ ^,^ ^20^ ^)^:

(5) $$D_{\text{eff}}={\left(\Sigma v_{i},{D_{i}}^{1/n}\right)}^{n}.$$

The parameter *n* describes the strength of the volume effect. In the literature three different *n* values have been reported that assume different volume dependence for rectal complications, with n=0.12,^(12)^ n=0.24,^(10)^ and n=0.746.^(9)^ All the parameters used in the calculations are shown in Table 2. The results of the simulations have been summarized as dose‐population histograms (DPH).^(7)^ Similar to a DVH, each point on the cumulative DPH represents the percentage of patients who receive at least a certain dose. Generally, dose in a DPH curve can have a broad meaning, including minimum dose to the GTV, effective dose to an OAR, such as in this paper, or any other dose chosen as a representative of outcome.

### D. Plan evaluation

The rectum is considered to be the limiting organ for prostate dose escalation. For 70 Gy/35 fx, as in our Reference Plan, the probability of rectal grade 2 bleeding, scored with the modified RTOG‐SOMA scale, is 12% to 14%.^(^ ^1^ ^,^ ^22^ ^)^ We assumed that patients in the high‐dose region of the DPH curve are at higher risk to develop complications. We therefore strengthened the acceptance criteria for dose escalation protocols. For each patient, we determined the $D_{\text{eff}}$ on the DPH curve that corresponded to 20% of patients in the Reference Plan as the dose leading to acceptable risk. For the Escalated Plan to be acceptable, the risk should be lower. In this study, less than 20% of patients treated with realignment must have their rectum $D_{\text{eff}}$ lower or equal to the risk level defined by the Reference Plan. This risk level is the baseline for acceptance of the Escalated Plan. Further, it connects our simulation results with the clinical consequences.

### E. Potential full 3D correction

In Chung et al.,^(8)^ the online correction protocol only uses one lateral EPI to correct the AP and SI geometric error. This allows for the realignment to be performed in a reasonable time frame (around 8 min). Considering that the lateral shift of the prostate should be small compared with AP and SI shifts, this procedure appears reasonable. However, the potential benefits of full 3D correction have to be explored.

A full 3D realignment protocol is currently used in our center. To evaluate biological consequences of this full 3D realignment quantitatively, we simulated reduction of uncertainties in all dimensions. However, because only a small number of patients have been treated to date with implanted fiducial markers at this center, the full 3D realignment data are presently insufficient to reliably establish uncertainties in 3D. Therefore, we manipulated the systematic and random uncertainties in the lateral direction to make geometric error reduction consistent with what was achieved for other directions. Instead of systematic uncertainty of 3.2 mm and random uncertainty of 2.2 mm (see Table 1), we assumed 0.5 mm and 1.1 mm, respectively. This simulation was performed for the Escalated Plan.

## III. RESULTS

### A. Tumor control

**Figure 1:**
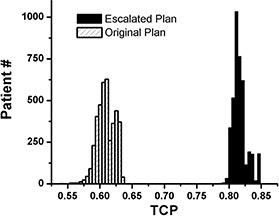
TCP histograms showing the Escalated Plan and the Reference Plan for all patient simulations

All 4000 (20 treatment plan×200 histories) virtual patients’ TCPs are shown in Fig. 1. The Escalated Plan average TCP is 0.817, with a standard deviation of 0.011. In comparison, the Reference Plan gives a mean TCP of 0.609 and a standard deviation of 0.014. This gain in tumor control is consistent with parameter values used in our calculations, specifically, $\gamma_{50}$ (see Table 2). Values of the normalized slope for prostate cancer reported in the literature vary with a range of 1.0 to 3.2^(23)^ (intermediate risk prostate cancer). The impact of different parameters has been investigated in our previous work.^(9)^ While dose escalation is always beneficial, the expected improvement in local control will of course be less if a shallower dose‐response is assumed.

### B. Rectal complications

**Figure 2:**
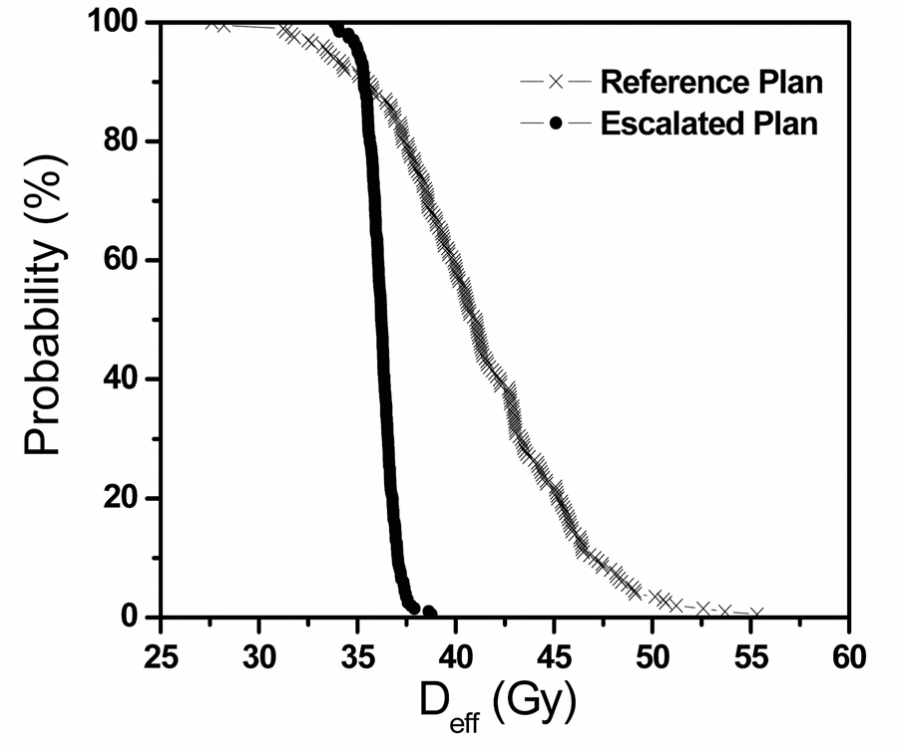
Rectal dose population histograms (DPH) for one of the 20 patients (n=0.746). The slope of the Reference Plan curve is broad while that for the Escalated Plan curve is steep. Beyond the crossing, the Escalated Plan gives lower dose to the rectum than the Reference Plan and vice versa.

The typical DPH curve for a single patient simulation is shown in Fig. 2. The vertical axis is the percentage of all 200 histories, and the horizontal axis is the rectum $D_{\text{eff}}$. The Reference Plan (70 Gy with 10‐mm margin and full uncertainty) leads to a broad distribution in $D_{\text{eff}}$. In contrast, the DPH for the Escalated Plan (78 Gy with 5‐mm margin and reduced uncertainty) is steeper than the Reference Plan. If one DPH was fully “contained” in another, the preference from a clinical perspective would be trivial. However, quite often the DPHs cross over at one point. The point at which two DPHs cross strongly depends on the value of the parameter *n* used in the $D_{\text{eff}}$ calculation. In the region where $D_{\text{eff}}$ is greater than the point of crossing, the Escalated Plan always gives lower rectum dose as compared with the Reference Plan and vice versa. If the point of crossing corresponds to a $D_{\text{eff}}$ of minor clinical consequence, that is, rectal toxicity, then the Escalated protocol would be superior and safer compared to the Reference Plan.

**Figure 3:**
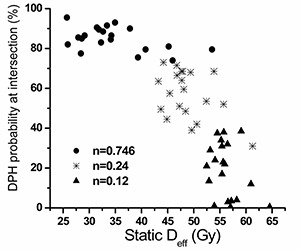
The DPH probability at intersection point versus static $D_{\text{eff}}$ plot for each patient with different *n* values. The horizontal axis is the static $D_{\text{eff}}$ of the Reference Plan. The vertical axis is the percentage in population.

Figure 3 shows the relationship between the location of crossing, presented as the vertical axis value of the probability in the DPH at which the two plans intersect, and the static $D_{\text{eff}}$ of each patient from the Reference Plan as an index. The static $D_{\text{eff}}$ is calculated without organ motion simulation. In other words, the dose to the rectum for every fraction is delivered as planned. As was addressed above, $D_{\text{eff}}$ changes with *n*. The graph shows the proportion of patients who would benefit if they were treated with the escalated dose, smaller margin, and patient realignment, compared to the treatment with full uncertainty. If the point of crossing falls on a clinically inconsequential $D_{\text{eff}}$, we can assume that no patient will be subjected to a higher risk of rectal toxicity compared to the Reference Plan. From the graph, the dose escalation protocol reduces rectal dose to a large proportion of patients with large $D_{\text{eff}}$, if we assume rectum behavior is more parallel, that is, n=0.746 ^(9)^ or n=0.24.^(10)^ However, if more serial behavior is assumed, n=0.12,^(12)^ some of the virtual patients have shown no obvious improvement, exceeding the 20% risk level.

### C. Look‐up plots for static $D_{\text{eff}}$ versus realistic $D_{\text{eff}}$

Look‐up plots for the relationship between the $D_{\text{eff}}$ from the planning DVH (static $D_{\text{eff}}$) and the realistic doses (blurred $D_{\text{eff}}$) to patient accounting for geometric uncertainty in treatment, are shown in Figs. 4(a), (b), and (c). The three possible parameters describing strength of volume effects were modeled for two prescription doses. The median $D_{\text{eff}}$ and 95% confidence limits derived from the 200 histories are shown for the entire dataset. Variation in planned $D_{\text{eff}}$ arises from patient anatomy differences. These graphs illustrate the relationship between the dose distribution calculated by the treatment‐planning system and what is actually delivered to the patient. These graphs also clearly show how potential benefits of applying tighter PTV margins and escalating the dose depend on the assumed strength of volume effects.

**Figure 4(a):**
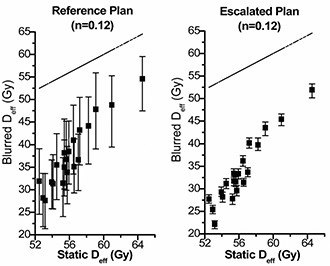
Static $D_{\text{eff}}$ of the Reference Plan versus the blurred $D_{\text{eff}}$ of the Reference Plan (left) and the Escalated Plan (right) for n=0.12. The blurred $D_{\text{eff}}$, due to geometric uncertainty associated in treatment, is shown as a median value and the 95% confidence limits. The dashed line shows equal static $D_{\text{eff}}$ and blurred $D_{\text{eff}}$ as a reference.

**Figure 4(b):**
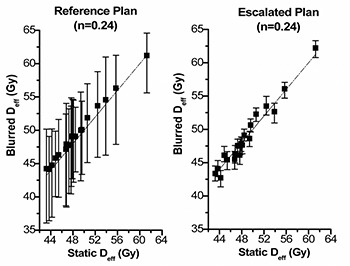
Static $D_{\text{eff}}$ of the Reference Plan versus the blurred $D_{\text{eff}}$ of the Reference Plan (left) and the Escalated Plan (right) for n=0.24. The blurred $D_{\text{eff}}$, due to geometric uncertainty associated in treatment, is shown as a median value and the 95% confidence limits. The dashed line shows equal static $D_{\text{eff}}$ and blurred $D_{\text{eff}}$ as a reference.

**Figure 4(c):**
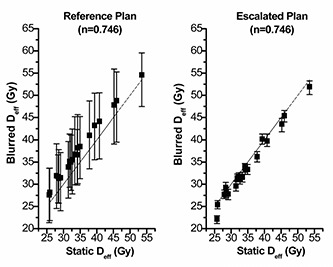
Static $D_{\text{eff}}$ of the Reference Plan versus the blurred $D_{\text{eff}}$ of the Reference Plan (left) and the Escalated Plan (right) for n=0.746. The blurred $D_{\text{eff}}$, due to geometric uncertainty associated in treatment, is shown as a median value and the 95% confidence limits. The dashed line shows equal static $D_{\text{eff}}$ and blurred $D_{\text{eff}}$ as a reference.

### D. Potential 3D full correction

**Figure 5:**
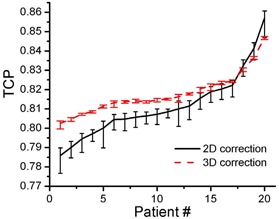
TCP graph showing mean values for 2D correction and potentially 3D full correction with the 95% confidence limits for the 20 patients

The results with additional lateral realignment show that the calculated average increase in the lowest TCP (considering the worst scenario) among the 20 patients is 0.83%, shown in Fig. 5. The $D_{\text{eff}}$ to rectum and bladder has no significant change with 3D full correction for all possible *n* values.

## IV. DISCUSSION

The intuitive thinking for isotoxicity or toxicity‐free dose escalation protocol is to reduce the geometric uncertainties, and then reduce the PTV margins. This has been pointed out by authors who presented results of prostate intensity‐modulated radiotherapy (IMRT) or stereotactic radiation therapy for prostate cancer.^(^ ^21^ ^,^ ^22^ ^,^ ^24^ ^–^ ^26^ ^)^ This drives image‐guided radiation therapy to be the next step in radiation therapy. Patient realignment using internal fiducial markers and EPI has proven to be a reliable approach. Uncertainty control by EPI applied to prostate IMRT has been reported in the literature.^(^ ^21^ ^,^ ^22^ ^)^ Dose escalation protocols were suggested with patient realignment and margin reduction being an integral part of the protocol.

### A. Geometric uncertainties

This work is intended to investigate the dose escalation protocol using Princess Margaret Hospital's previously published online patient realignment protocol.^(8)^ Therefore, the geometric uncertainties for patient realignment are taken from their publication.^(8)^ The geometric uncertainties for current treatment procedure, no realignment, are taken from the study of van Herk et al.^(7)^ The data are believed to represent the clinical situation at our center because we have the same patient management: full bladder without rectum balloon. The magnitude of geometric uncertainties will affect the simulation results. However, since we follow the clinical data, our results reveal the clinical consequences.

In this study, we simulated the organ motion without organ deformation. The rectum and the bladder are considered to be the most deformable organs instead of the prostate. If the organ deformation had been considered, the biological consequences of the dose escalation protocol may have been affected. Incorporating organ deformation is planned for our future studies.

### B. Patient anatomy

For dose to critical organs, there are two factors that contribute to the dose variation among the population: geometric errors, which will “blur” the dose distribution for a single patient, and the patient's anatomy. Some patients may receive higher doses to a critical organ because of the relative positions of the critical organ and the prostate. This has been offset to some extent by choosing alternative beam arrangements, for example, IMRT versus four‐field. In clinical practice, plan acceptance depends on the dose distribution calculated for the planning CT. In this study, we calculated look‐up plots that allow us to estimate more realistic dose variation when uncertainties are full (no realignment) and reduced (patient realigned prior to every fraction). These look‐up plots can be used to identify patients who may be suitable for dose escalations without excess risk of rectal complications.

### C. Rectal complications

In our study, we considered gastrointestinal complications only. Genito‐urinary (GU) complications were not investigated because they are difficult to analyze, hematuria may originate from either the bladder or the urethra, and there is no consistent bladder or urethral DVH correlation for GU complication. Because of the reduced PTV margin, better sparing of surrounding organs, including the bladder, can be achieved. Although quantification of biological consequences of this margin reduction in terms of GU complications is associated with significant uncertainties, one would still speculate that there is an advantage in reducing radiation dosage received by the bladder. In terms of erectile dysfunction, again, one would expect an advantage with a tighter margin and thus less radiation dosage to the penile bulb, and perhaps better preservation of sexual function. But without extensive supporting data showing there is a strong correlation of penile bulb dosimetry and erectile dysfunction, we did not analyze this effect, although this remains a very interesting research question.

For rectal complications, we use the $D_{\text{eff}}$ as our evaluation scale. The $D_{\text{eff}}$ was calculated by reducing rectal DVH to one value. In this procedure an *n* factor was used, which describes the strength of the rectum volume dependence. A larger *n* value corresponds to more parallel dose‐volume dependence. However, currently there is no consensus about this value.

The value of the parameter describing the strength of volume effects (n=0.12 ^(12)^) reported by Burman et al. was obtained from the TD5/5 and TD50/5 data given by Emami et al.^(11)^ Due to the lack of clinical evidence for partial organ tolerance doses, expert opinion was used. Burman et al. acknowledged that “To determine the volume dependence parameter, *n*, for organs with insufficient data, a best clinical estimate was made by a group of investigators.” Therefore, n=0.12 originates from professional opinion rather than dose‐volume based clinical data.

The n=0.746 ^(9)^ reported by the M.D. Anderson hospital group was based on clinically observed complications and planning DVHs. The study group comprised 128 patients, but they excluded patients with hemorrhoids to obtain the above *n* factor, a further reduction to 84 patients. The prescription dose was the same for all patients: 46 Gy with a four‐field box technique followed by a six‐field arrangement to boost the total target dose to 78 Gy.

The latest data, n=0.24,^(10)^ are given by a multicenter study performed in Italy. The patient population was 547. Different prescription doses were used, ranging from 64 Gy to 79.2 Gy. Presently, this is the most broadly based study both in terms of sample size and variation in dose‐volume distributions.

From the clinical data, it appears that the rectum behavior is not as serial as we long believed, suggesting that an n=0.12 value may not be appropriate. Some hints for this were provided by clinical practice using 3D CRT. The Memorial Sloan‐Kettering Cancer Center ^(27)^ reported that with a prescription dose of 81 Gy and a 1.0‐cm margin PTV, except at the prostate—rectum interface, where a 0.6‐cm margin was used, rectal grade 2 or higher (RTOG scale) complication rate was 16.5%. In the M.D. Anderson report,^(28)^ a 78 Gy prescription dose with a tighter margin was used: “the block edge was placed 1.25 cm to 1.5 cm around the CTV in the anterior and inferior directions and 0.75 cm to 1.0 cm in the posterior and superior directions.” The rectal grade 2 or higher (RTOG scale) complication rate was 21%. This clinical evidence shows that there is no dramatic increase in rectal complications as the serial model predicted.

According to the Italian multicentric analysis data^(10)^ for rectum (n=0.24), dose escalation with patient realignment not only improves the local control, but also gives a lower probability of rectal complications; see Fig. 3.

Margin reduction and dose escalation, according to our results, can be safely achieved with realignment. However, rectal volume effects put strong constraints on this. Therefore, prostate dose escalation studies have to include collection of full dose‐volume data for OARs and possibly dose‐rectal wall data.

### D. Potential full 3D correction

From Fig. 5, for most of the patients, a full 3D correction gives better local control than 2D correction. However, in terms of average TCP, the increase is less than 1%. For $D_{\text{eff}}$ to rectum, the 3D correction does not show an advantage compared to the 2D correction. Considering the extra workload of lateral geometric uncertainty correction, correction of lateral geometric uncertainty may provide little clinical benefit.

## V. CONCLUSION

According to our analysis of the rectum dose and assuming a parallel behavior for rectal complications for patients treated with prostate fields only, prescription dose can be escalated from 70 Gy/35 fx to 78 Gy/39 fx with reduced 3D PTV margin from 10 mm to 5 mm if patient repositioning by EPI and internal fiducial markers is used. Current data support more parallel response for rectal bleeding. If this is confirmed, further escalation might be achievable. Look‐up plots for static $D_{\text{eff}}$ versus realistic $D_{\text{eff}}$ were generated. They can be used to improve the clinical decision process for plan evaluation and acceptance. Full 3D correction is not substantially superior to the 2D correction.

## ACKNOWLEDGMENT

We thank Tim Craig for help with organ motion simulation. Special thanks to Ken Yuen for help with the treatment‐planning check.
